# Supplementary material for: Multi‐dataset identification of innovative feature genes and molecular mechanisms in keratoconus
Source: J Cell Mol Med. 2024 Sep 19;28(18):e70079. doi: 10.1111/jcmm.70079 (PMC11412914; doi:10.1111/jcmm.70079)
Supplement: Supplementary file 2 — Material S2. [file JCMM-28-e70079-s002.zip › 7.GSEA analysis/1.ARL11/KEGG_PANCREATIC_CANCER.html]

Details for gene set KEGG\_PANCREATIC\_CANCER[GSEA]

|  || Dataset | ARL11.group.cls#h\_versus\_l |
| Phenotype | group.cls#h\_versus\_l |
| Upregulated in class | h |
| GeneSet | KEGG\_PANCREATIC\_CANCER |
| Enrichment Score (ES) | 0.18517871 |
| Normalized Enrichment Score (NES) | 0.7940938 |
| Nominal p-value | 0.725 |
| FDR q-value | 1.0 |
| FWER p-Value | 1.0 |
Table: GSEA Results Summary

  

Fig 1: Enrichment plot: KEGG\_PANCREATIC\_CANCER      
 Profile of the Running ES Score & Positions of GeneSet Members on the Rank Ordered List

  

| SYMBOL | TITLE | RANK IN GENE LIST | RANK METRIC SCORE | RUNNING ES | CORE ENRICHMENT || 1 | ARHGEF6 | na | 432 | 0.494 | 0.0782 | Yes |
| 2 | PIK3R3 | na | 2180 | 0.284 | 0.0799 | Yes |
| 3 | TGFBR2 | na | 2250 | 0.279 | 0.1292 | Yes |
| 4 | PIK3CG | na | 2870 | 0.247 | 0.1566 | Yes |
| 5 | RAC3 | na | 4612 | 0.179 | 0.1392 | Yes |
| 6 | BRCA2 | na | 5798 | 0.142 | 0.1312 | Yes |
| 7 | PIK3R1 | na | 6079 | 0.135 | 0.1479 | Yes |
| 8 | TGFB2 | na | 7601 | 0.100 | 0.1224 | Yes |
| 9 | CDKN2A | na | 8387 | 0.085 | 0.1153 | Yes |
| 10 | IKBKB | na | 8698 | 0.079 | 0.1209 | Yes |
| 11 | PIK3R2 | na | 8783 | 0.078 | 0.1329 | Yes |
| 12 | SMAD2 | na | 8821 | 0.078 | 0.1461 | Yes |
| 13 | VEGFC | na | 8842 | 0.077 | 0.1597 | Yes |
| 14 | AKT2 | na | 9348 | 0.070 | 0.1579 | Yes |
| 15 | TP53 | na | 9447 | 0.068 | 0.1675 | Yes |
| 16 | PIK3R5 | na | 10118 | 0.059 | 0.1590 | Yes |
| 17 | RALGDS | na | 10287 | 0.056 | 0.1645 | Yes |
| 18 | SMAD4 | na | 10504 | 0.054 | 0.1682 | Yes |
| 19 | BRAF | na | 10628 | 0.052 | 0.1742 | Yes |
| 20 | KRAS | na | 10630 | 0.052 | 0.1838 | Yes |
| 21 | EGFR | na | 11115 | 0.046 | 0.1783 | Yes |
| 22 | MAPK9 | na | 11167 | 0.046 | 0.1852 | Yes |
| 23 | PGF | na | 12205 | 0.034 | 0.1615 | No |
| 24 | PIK3CA | na | 13512 | 0.023 | 0.1281 | No |
| 25 | RALBP1 | na | 13546 | 0.023 | 0.1313 | No |
| 26 | EGF | na | 13590 | 0.022 | 0.1341 | No |
| 27 | VEGFB | na | 14052 | 0.018 | 0.1242 | No |
| 28 | SMAD3 | na | 14170 | 0.017 | 0.1240 | No |
| 29 | PLD1 | na | 14860 | 0.012 | 0.1063 | No |
| 30 | CHUK | na | 15127 | 0.010 | 0.1005 | No |
| 31 | MAPK10 | na | 15299 | 0.009 | 0.0973 | No |
| 32 | STAT1 | na | 15344 | 0.009 | 0.0976 | No |
| 33 | ERBB2 | na | 16056 | 0.003 | 0.0777 | No |
| 34 | RB1 | na | 16201 | 0.002 | 0.0740 | No |
| 35 | RAC1 | na | 16390 | 0.001 | 0.0687 | No |
| 36 | E2F2 | na | 16509 | 0.000 | 0.0654 | No |
| 37 | CCND1 | na | 16546 | -0.000 | 0.0643 | No |
| 38 | RALA | na | 17494 | -0.007 | 0.0382 | No |
| 39 | E2F1 | na | 17721 | -0.008 | 0.0333 | No |
| 40 | AKT1 | na | 18085 | -0.011 | 0.0248 | No |
| 41 | CDC42 | na | 18361 | -0.013 | 0.0192 | No |
| 42 | RALB | na | 18562 | -0.014 | 0.0160 | No |
| 43 | CDK4 | na | 18688 | -0.015 | 0.0151 | No |
| 44 | RAC2 | na | 18868 | -0.016 | 0.0129 | No |
| 45 | JAK1 | na | 19011 | -0.017 | 0.0119 | No |
| 46 | BAD | na | 19342 | -0.019 | 0.0060 | No |
| 47 | TGFBR1 | na | 19919 | -0.023 | -0.0064 | No |
| 48 | CASP9 | na | 20358 | -0.026 | -0.0142 | No |
| 49 | MAPK3 | na | 22614 | -0.046 | -0.0707 | No |
| 50 | IKBKG | na | 22799 | -0.048 | -0.0672 | No |
| 51 | RAF1 | na | 23336 | -0.054 | -0.0728 | No |
| 52 | PIK3CB | na | 23818 | -0.060 | -0.0757 | No |
| 53 | MAPK8 | na | 24525 | -0.070 | -0.0832 | No |
| 54 | MAPK1 | na | 24626 | -0.071 | -0.0731 | No |
| 55 | CDK6 | na | 24642 | -0.071 | -0.0604 | No |
| 56 | E2F3 | na | 25055 | -0.077 | -0.0582 | No |
| 57 | STAT3 | na | 25255 | -0.079 | -0.0494 | No |
| 58 | TGFB1 | na | 25459 | -0.082 | -0.0402 | No |
| 59 | PIK3CD | na | 25759 | -0.087 | -0.0329 | No |
| 60 | ARAF | na | 26195 | -0.093 | -0.0283 | No |
| 61 | NFKB1 | na | 26767 | -0.104 | -0.0258 | No |
| 62 | TGFA | na | 27293 | -0.114 | -0.0199 | No |
| 63 | AKT3 | na | 27547 | -0.120 | -0.0052 | No |
| 64 | RAD51 | na | 27599 | -0.121 | 0.0155 | No |
| 65 | VEGFA | na | 28646 | -0.148 | 0.0125 | No |
| 66 | BCL2L1 | na | 29160 | -0.161 | 0.0272 | No |
| 67 | RELA | na | 29186 | -0.162 | 0.0562 | No |
| 68 | MAP2K1 | na | 31070 | -0.223 | 0.0428 | No |
| 69 | TGFB3 | na | 33262 | -0.342 | 0.0423 | No |
Table: GSEA details [plain text format]

  

Fig 2: KEGG\_PANCREATIC\_CANCER      
 Blue-Pink O' Gram in the Space of the Analyzed GeneSet

  

Fig 3: KEGG\_PANCREATIC\_CANCER: Random ES distribution      
 Gene set null distribution of ES for **KEGG\_PANCREATIC\_CANCER**
